# Usefulness of qSOFA and ECOG Scores for Predicting Hospital Mortality in Postsurgical Cancer Patients without Infection

**DOI:** 10.1155/2019/9418971

**Published:** 2019-05-02

**Authors:** Silvio A. Ñamendys-Silva, Emerson Joachin-Sánchez, Aranza Joffre-Torres, Bertha M. Córdova-Sánchez, Guadalupe Ferrer-Burgos, Octavio González-Chon, Angel Herrera-Gomez

**Affiliations:** ^1^Department of Critical Care Medicine, Instituto Nacional de Cancerología, Mexico City, Mexico; ^2^Department of Critical Care Medicine, Medica Sur Clinic & Foundation, Mexico City, Mexico; ^3^Department of Critical Care Medicine, Instituto Nacional de Ciencias Médicas y Nutrición Salvador Zubirán, Mexico City, Mexico

## Abstract

**Background:**

The quick sequential organ failure assessment (qSOFA) and the Eastern Cooperative Oncologic Group (ECOG) scale are simple and easy parameters to measure because they do not require laboratory tests. The objective of this study was to compare the discriminatory capacity of the qSOFA and ECOG to predict hospital mortality in postsurgical cancer patients without infection.

**Methods:**

During the period 2013–2017, we prospectively collected data of all patients without infection who were admitted to the ICU during the postoperative period, except those who stayed in the ICU for <24 hours or patients under 18 years. The ECOG score during the last month before hospitalization and the qSOFA performed during the first hour after admission to the intensive care unit (ICU) were collected. The primary outcome for this study was the in-hospital mortality rate.

**Results:**

A total of 315 patients were included. The ICU and hospital mortality rates were 6% and 9.2%, respectively. No difference was observed between the qSOFA [AUC=0.75 (95% CI = 0.69-0.79)] and the ECOG scores [AUC=0.68 (95%CI =0.62-0.73)] (p=0.221) for predicting in-hospital mortality. qSOFA greater than 1 predicted in-hospital mortality with a high sensitivity (100%) but low specificity (38.8%); positive predictive value of 26.3% and negative predictive value of 93.1% compared to 74.4% of specificity, 55.1% of sensitivity%; positive predictive value of 18% and negative predictive value of 94.2% for an ECOG score greater than 1. Multivariable Cox regression analysis identified two independent predicting factors of in-hospital mortality, which included ECOG score during the last month before hospitalization (HR: 1.46; 95 % CI: 1.06-2.00); qSOFA calculated in the first hours after ICU admission (OR: 3.17; 95 % CI: 1.79–5.63).

**Conclusion:**

No difference was observed between the qSOFA and ECOG for predicting in-hospital mortality. The qSOFA score performed during the first hour after admission to the ICU and ECOG scale during the last month before hospitalization were associated with in-hospital mortality in postsurgical cancer patients without infection. The qSOFA and ECOG score have a potential to be included as early warning tools for hospitalized postsurgical cancer patients without infection.

## 1. Introduction

The introduction of new treatments for cancer and advances in intensive care have improved the outcomes of critically ill cancer patients. Patients with cancer may require admission to the intensive care unit (ICU) after surgery [1]. The quick sequential organ failure assessment (qSOFA) [2] and the Eastern Cooperative Oncologic Group (ECOG) scale [3] are simple and easy parameters to measure because they do not require laboratory tests. The qSOFA consists of three clinical elements, hypotension (systolic blood pressure ≤100 mmHg), tachypnea (respiratory rate ≥22 minute^−1^), and alteration in mental status (Glasgow Coma Score ≤13 points) (total score ranges between 0 and three) [2]. The qSOFA was derived from data of symptomatic patients; thus, it is not a screening tool for sepsis [4]. Patients with acute medical illness, such as acute coronary syndrome, hypovolemic shock, or trauma, may have a qSOFA ≥ 2. The qSOFA has been used to predict mortality in patients without suspected infection [5, 6]. The ECOG is used by oncohematologists and intensivists for decision making for cancer patients [1, 7–9]. The performance status impairment classified according to the ECOG has prognostic value in general critically ill patients [7] and critically ill cancer patients [8]. The ECOG has six categories (total score ranges between 0 and five):

- Score of 0: indicating that the patient is fully active, able to carry on all pre-disease performance without restriction
- Score of 1: indicating restriction in physically strenuous activity, but the patient is still ambulatory and able to carry out work of a light or sedentary nature (e.g., light house work; office work)
- Score of 2: indicating that the patient is ambulatory and capable of all self-care but unable to carry out any work activities. Up and about more than >50 % of waking hours
- Score of 3: indicating that the patient is capable of only limited self-care and is confined to the bed or chair more than >50 % of waking hours
- Score of 4: indicating that the patient is completely disabled, cannot carry on any self-care: totally confined to the bed or chair
- Score of 5: indicating death

The performant status impairment in the week before hospital admission has been associated with increased hospital mortality in the critically ill patients [7].

The objective of this study was to compare the discriminatory capacity of the qSOFA [2] and ECOG [3] to predict hospital mortality in postsurgical cancer patients without infection.

## 2. Methods

This observational study was performed in the ICU of the Instituto Nacional de Cancerología (INCan), Mexico City. The Bioethics Committee of INCan approved this study, and the need for informed consent was waived (Rev/03/2013). During the period 2013-2017, we collected data of all patients without infection who were admitted to the ICU during the postoperative period, except those who stayed in the ICU for <24 hours or patients under 18 years. Demographic and clinical data were collected during the first day of the ICU stay, including the ECOG score during the last month before hospitalization, type of tumor, cancer status, need for mechanical ventilation (MV), length of invasive MV, length of stay in the ICU, length of stay in the hospital, and ICU and in-hospital mortality. The qSOFA was performed during the first hour after admission to the ICU. The qSOFA was determined by assigning one point for each of the following variables: Glasgow coma scale <15 before surgery, systolic blood pressure ≤100 mm Hg, or respiratory rate ≥22/min [2]. Each patient's length of stay in the hospital was measured based on the number of days between their admission and discharge from the INCan. The disease status was categorized into the following: recently diagnosed (prior to treatment or administration of first line of treatment), active disease (disease progression or during second- and third-line treatment), and complete remission.

### 2.1. Statistical Analysis

The primary outcome (the dependent variable) for this study was the in-hospital mortality rate. Continuous variables are expressed as the means ± standard deviation or as medians and interquartile ranges for skewed distributions. Categorical variables are expressed as percentages. To assess the performances of the qSOFA and the ECOG scores to predict in-hospital mortality, we calculated the sensitivity, specificity, negative predictive value, positive predictive value, positive likelihood ratio, and negative likelihood ratio. The area under the receiver operating characteristic curve (AUC) was used to evaluate the ability of the qSOFA and ECOG scores to discriminate between patients who lived and those who died. Comparison of the AUC was performed using the methodology suggested by Hanley and McNeil [10]. Cox proportional hazards univariate and multivariate analyses (forward selection) were used to identify factors with potential prognostic significance for in-hospital mortality. The final Cox model was assessed for potential interactions. The results were reported using hazard ratios (HRs) and the corresponding 95% confidence intervals (95% CIs). Survival curves were estimated using the Kaplan-Meier method. A two-sided p value <0.05 was used to determine statistical significance.

## 3. Results

**Figure 1:**
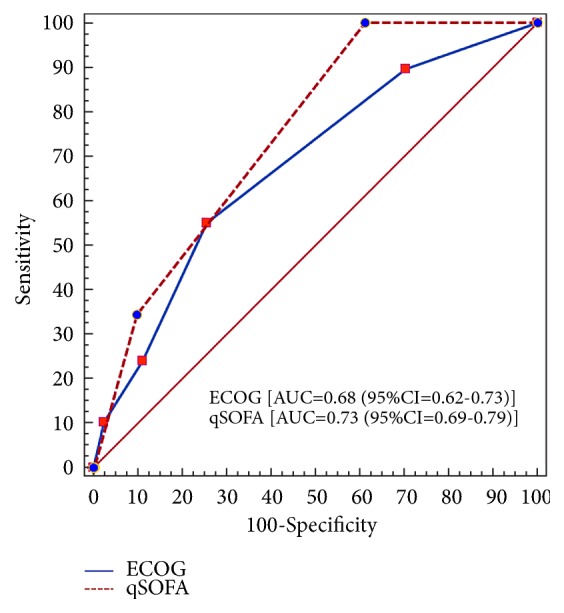
Comparisons of the areas under the receiver operating characteristic curves for the prediction of in-hospital mortality of the quick sequential organ failure assessment (qSOFA) and the Eastern Cooperative Oncologic Group (ECOG) scale.

**Figure 2:**
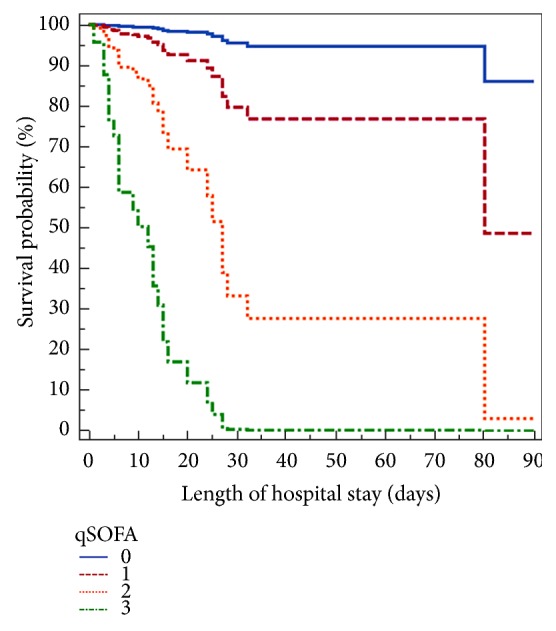
Survival probabilities in postsurgical cancer patients without infection, according to the quick sequential organ failure assessment (qSOFA) scale.

**Figure 3:**
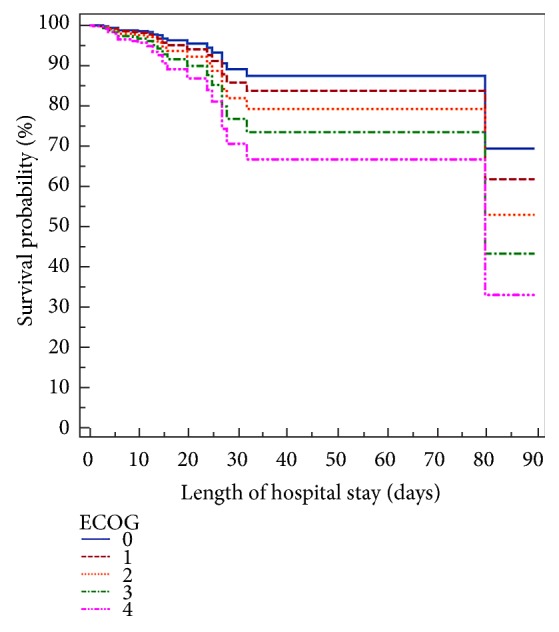
Survival probabilities in postsurgical cancer patients without infection, according to the Eastern Cooperative Oncologic Group (ECOG) scale.

A total of 315 patients were included. The mean age of the patients was 50.6 ± 15.9 years, and 59% (186) were female. There were 195 patients (61.9%) who required invasive MV during their stay in the ICU, with a median duration of two days (1-4 days), and the median length of stay in the ICU was 2 days (1-4 days). Sixty-eight (21.6%) patients had a gynecological malignancy, while the other most common primary cancer sites were the gastrointestinal (16.2%) and sarcoma (14.3%). In terms of cancer status, 50.8% were newly diagnosed, 48.8% had disease progression, and 0.3% had complete remission of disease.  Table 1 reports the main clinical characteristics. The ICU and hospital mortality rates were 6% and 9.2%, respectively. For patients with qSOFA scores less than 2, the hospital mortality rate was 7.36% vs 35.7% for patients with a qSOFA score of 2 or higher (absolute difference, 28.3%; 95% CI, 13%-47.7%, p<0.001). For patients with ECOG scores less than 2, the hospital mortality rate was 6.1% vs 21.9% for patients with a ECOG score of 2 or higher (absolute difference, 15.8%; 95% CI, 6.8%-26.1%, p<0.001). No difference was observed between the qSOFA [AUC=0.75 (95% CI = 0.69-0.79)] and the ECOG scores [AUC=0.68 (95%CI =0.62-0.73)] (p=0.221) for predicting in-hospital mortality (Figure 1).  Table 2 reports the sensitivity, specificity, positive predictive value, and negative predictive value for the qSOFA and ECOG scores for predicting in-hospital mortality. qSOFA greater than 1 predicted in-hospital mortality with a high sensitivity (100%) but low specificity (38.8%); positive predictive value of 26.3% and negative predictive value of 93.1% compared to 74.4% of specificity, 55.1% of sensitivity%; positive predictive value of 18% and negative predictive value of 94.2% for an ECOG score greater than 1 (Table 2). Multivariable Cox regression analysis identified two independent predicting factors of in-hospital mortality, which included ECOG score during the last month before hospitalization (HR: 1.46; 95 % CI: 1.06-2.00) and qSOFA calculated in the first hours after ICU admission (HR: 3.17; 95 % CI: 1.79–5.63) (Table 3). Survival probabilities in postsurgical cancer patients without infection, according to the qSOFA and ECOG scale, are shown in Figures 2 and 3.

## 4. Discussion

The main findings of this study are that no difference was observed between the qSOFA and ECOG for predicting in-hospital mortality. Both the qSOFA and ECOG scale demonstrated a poor to fair level of discrimination for in-hospital mortality. The qSOFA has been used to predict mortality in patients without suspected infection [5, 6]. Singer et al. [5] reported the utility of qSOFA for assessing the outcome of adult emergency department (ED) patients without suspected infection. The qSOFA score at the time of ED admission (within 2 minutes or less) demonstrated an AUC=0.70 (95% CI=0.65-0.74), suggesting fair accuracy for mortality prediction. Jawa et al. [6] reported the ability of the qSOFA to predict outcomes in blunt trauma patients presenting to the ED. The qSOFA score calculated from the initial vital signs in the ED demonstrated an AUC of 0.73 [95% CI 0.69-0.76]. Similarly, in our cohort of critically ill cancer patients without infection the qSOFA score demonstrated a fair level of discrimination for in-hospital mortality prediction. The performance status impairment classified according to the ECOG has prognostic value in general critically ill patients [7] and critically ill cancer patients [8]. The ECOG scale could account for factors that cannot be accounted for by critical care severity scores. Park et al. [9] reported a significant trend for increasing hospital mortality as the ECOG score became higher. The qSOFA is not part of the new sepsis definitions; a critically ill patient or cancer patient may have a qSOFA ≥2 without infection or sepsis: for example, acute coronary syndrome, hypovolemic shock, or trauma. The qSOFA and ECOG score of 2 or higher could identify the critical point at which critically ill cancer patients without infection exhibit the highest risk of death during hospitalization. The qSOFA and ECOG scores are simple and easy to measure; they can be used as generic tools to predict clinically important outcomes for critically ill cancer patients likely to be admitted to the ICU regardless of whether infection is suspected. A strength of this study is that it presents the outcomes of postsurgical cancer patients without infection admitted to ICU. To the best of our knowledge, this study is the first to compare the predictive accuracy of the qSOFA and ECOG scales to predict the outcomes of critically ill cancer patients without infection. However, our study has some limitations in that it only included cancer patients without infection who were admitted to the ICU during the postoperative period and represents the experience of a single center.

## 5. Conclusion

No difference was observed between the qSOFA and ECOG for predicting in-hospital mortality. The qSOFA score performed during the first hour after admission to the ICU and ECOG scale during the last month before hospitalization were associated with in-hospital mortality in postsurgical cancer patients without infection. The qSOFA and ECOG score have a potential to be included as early warning tools for hospitalized postsurgical cancer patients without infection.

## Figures and Tables

**Table 1:** Clinical characteristics of critically ill patients without infection who were admitted to the ICU during the postoperative period (n = 315).

Characteristics	Finding
Age, years, mean ± SD	50.6 ± 15.9
Gender (female), n (%)	186(59)
Length of stay in ICU (days), median (IQR)	2 (1–4)
Length of stay in the hospital (days), median (IQR)	8(6-15)
Quick sequential organ failure assessment (qSOFA), n(%)	
qSOFA=0	111(35.2)
qSOFA=1	166(52.7)
qSOFA=2	37(11.3)
qSOFA=3	1(0.3)
Eastern Cooperative Oncologic Group ( ECOG), n (%)	
ECOG=0	88(27.9)
ECOG=1	138(43.8)
ECOG=2	50(15.9)
ECOG=3	29(9.2)
ECOG=4	10(3.2)
ICU mortality, n (%)	19(6)
Hospital mortality, n (%)	29 (9.2)

**Table 2:** Sensitivity, specificity, positive predictive value, negative predictive value, positive likelihood ratio, and negative likelihood ratio for quick sequential organ failure assessment (qSOFA) and Eastern Cooperative Oncologic Group (ECOG) for in-hospital mortality.

Finding	qSOFA				ECOG			
	>0	>1	>2	>3	>0	>1	>2	>3
Sensitivity, %	100.0	34.4	0	0	89.6	55.1	24.1	10.3
Specificity, %	38.8	90.21	99.6	100.0	29.7	74.4	88.8	97.5
Positive predictive value, %	14.2	26.3	0	0	11.5	18.0	17.9	30.0
Negative predictive value, %	100	93.1	90.8	90.8	96.6	94.2	92.0	91.5
Positive likelihood ratio	1.63	3.52	0.0	0.0	1.2	2.16	2.16	4.2
Negative likelihood ratio	0.0	0.73	1.00	1.00	0.35	0.60	0.85	0.23

**Table 3:** Univariable and multivariable analysis of factors associated with in-hospital mortality.

Variable	Hazard ratio	95% CI	p	Hazard ratio	95% CI	p
	Univariate			Multivariate		
Age, years	1.02	0.99-1.04	0.062			
Gender, female	1.05	0.50-2.22	0.881			
ECOG, points	1.60	1.15-2.21	0.004	1.46	1.06-2.00	0.018
qSOFA, points	3.14	1.86-5.31	<0.001	3.17	1.79-5.63	<0.001
Length of stay in the ICU, days	0.95	0.87-1.04	0.301			

Abbreviations: CI=confidence interval, ICU=Intensive Care Unit, ECOG=Eastern Cooperative Oncology Group, and qSOFA= quick sequential organ failure assessment.

## Data Availability

The data used to support the findings of this study are available from the corresponding author upon request.

## Abbreviations

qSOFA: Quick sequential organ failure assessment
ECOG: Eastern Cooperative Oncologic Group
ICU: Intensive Care Unit
INCan: Instituto Nacional de Cancerología
MV: Mechanical ventilation
AUC: Area under the receiver operating characteristic curve
HR: Hazard ratio
ED: Emergency department.
